# Effects of positive end-expiratory pressure strategy in supine and prone position on lung and chest wall mechanics in acute respiratory distress syndrome

**DOI:** 10.1186/s13613-018-0434-2

**Published:** 2018-09-10

**Authors:** Mehdi Mezidi, Francisco José Parrilla, Hodane Yonis, Zakaria Riad, Stephan H. Böhm, Andreas D. Waldmann, Jean-Christophe Richard, Floriane Lissonde, Romain Tapponnier, Loredana Baboi, Jordi Mancebo, Claude Guérin

**Affiliations:** 1 Service de Réanimation Médicale, Hôpital de la Croix-Rousse, Hospices Civils de Lyon, Lyon, France; 2 Université de Lyon, Lyon, France; 3 Intensive Care Unit, Sant Pau Hospital, Barcelona, Spain; 4 Department of Anesthesiology and Intensive Care Medicine, Rostock University Medical Center, Schillingallee 35, 18057 Rostock, Germany; 5 Swisstom AG, Lanquart, Switzerland; 6 Department of Pneumology and Critical Care Medicine, Cologne-Merheim Hospital, Kliniken der Stadt Koln gGmbH, Witten/Herdecke University Hospital, Ostmerheimer Strasse 200, 51109 Cologne, Germany; 7 INSERM 955, Créteil, France

**Keywords:** Acute respiratory distress syndrome, Prone position, Positive end-expiratory pressure, Esophageal pressure, Electrical impedance tomography, Transpulmonary pressure

## Abstract

**Background:**

In acute respiratory distress syndrome (ARDS) patients, it has recently been proposed to set positive end-expiratory pressure (PEEP) by targeting end-expiratory transpulmonary pressure. This approach, which relies on the measurement of absolute esophageal pressure (Pes), has been used in supine position (SP) and has not been investigated in prone position (PP). Our purposes were to assess Pes-guided strategy to set PEEP in SP and in PP as compared with a PEEP/FIO_2_ table and to explore the early (1 h) and late (16 h) effects of PP on lung and chest wall mechanics.

**Results:**

We performed a prospective, physiologic study in two ICUs in university hospitals on ARDS patients with PaO_2_/FIO_2_ < 150 mmHg. End-expiratory Pes (Pes,ee) was measured in static (zero flow) condition. Patients received PEEP set according to a PEEP/F_I_O_2_ table then according to the Pes-guided strategy targeting a positive (3 ± 2 cmH_2_O) static end-expiratory transpulmonary pressure in SP. Then, patients were turned to PP and received same amount of PEEP from PEEP/F_I_O_2_ table then Pes-guided strategy. Respiratory mechanics, oxygenation and end-expiratory lung volume (EELV) were measured after 1 h of each PEEP in each position. For the rest of the 16-h PP session, patients were randomly allocated to either PEEP strategy with measurements done at the end. Thirty-eight ARDS patients (27 male), mean ± SD age 63 ± 13 years, were included. There were 33 primary ARDS and 26 moderate ARDS. PaO_2_/FIO_2_ ratio was 120 ± 23 mmHg. At same PEEP/FIO_2_ table-related PEEP, Pes,ee averaged 9 ± 4 cmH_2_O in both SP and PP (*P* = 0.88). With PEEP/F_I_O_2_ table and Pes-guided strategy, PEEP was 10 ± 2 versus 12 ± 4 cmH_2_O in SP and 10 ± 2 versus 12 ± 5 cmH_2_O in PP (PEEP strategy effect *P* = 0.05, position effect *P* = 0.96, interaction *P* = 0.96). With the Pes-guided strategy, chest wall elastance increased regardless of position. Lung elastance and transpulmonary driving pressure decreased in PP, with no effect of PEEP strategy. Both PP and Pes-guided strategy improved oxygenation without interaction. EELV did not change with PEEP strategy. At the end of PP session, respiratory mechanics did not vary but EELV and PaO_2_/F_I_O_2_ increased while PaCO_2_ decreased.

**Conclusions:**

There was no impact of PP on Pes measurements. PP had an immediate improvement effect on lung mechanics and a late lung recruitment effect independent of PEEP strategy.

**Electronic supplementary material:**

The online version of this article (10.1186/s13613-018-0434-2) contains supplementary material, which is available to authorized users.

## Background

With the currently used ventilator supportive management, the mortality of acute respiratory distress syndrome (ARDS) is still 30–40% [1]. Setting lower tidal volumes (VT) has been shown to improve survival by preventing further lung damage from excessive stress and strain [2]. Setting positive end-expiratory pressure (PEEP) was suggested as early as ARDS was described [3]. Three large trials failed to demonstrate survival benefit of using higher versus lower PEEP [4–6], but meta-analysis suggested a small but statistically significant benefit favoring of higher PEEP in severe ARDS [7].

Talmor and colleagues proposed to set PEEP by using end-expiratory transpulmonary pressure obtained by subtracting absolute esophageal pressure (Pes), a surrogate of pleural pressure, from airway pressure (Paw) at end-expiration. They proposed to increase PEEP in order to make end-expiratory transpulmonary pressure positive, and found marked physiologic benefits from this strategy [8]. While these results are awaiting confirmation from a large multicenter trial just completed [9], Pes monitoring has experienced a growing interest for the recent years [10].

Delivering lung protective ventilation in prone position (PP) in ARDS patients has been shown to improve survival [11–13] and is recommended in severe cases [14, 15]. Given that proning relieves the weight of mediastinum from the spinal parts of the lungs [16], the accuracy of Pes to reflect pleural pressure would be improved in PP as compared to supine (SP), as suggested recently in healthy patients under general anesthesia [17]. Accordingly, the measurement of end-expiratory transpulmonary pressure could be more relevant, in PP than in SP.

With this reasoning in mind, we underwent the present study to test the hypothesis that the static end-expiratory absolute Pes value (Pes,ee) would be lower in PP than in SP in ARDS patients. If this was true, this lower PEEP should be set in PP than in SP for a given static end-expiratory transpulmonary pressure (P_L_,ee). Our secondary objective was to explore the early and late effects of a PP session on arterial blood gas, lung and chest wall mechanics and regional ventilation according to the PEEP strategy.

## Methods

### Study design and population

Adult ARDS patients with PaO_2_/F_I_O_2_ ≤ 150 mmHg under invasive mechanical ventilation including PEEP ≥ 5 cmH_2_O and VT = 6 mL/kg predicted body weight were included in two academic medical intensive care units (Additional file 1: Figure S1).

### Mechanical ventilation

Patients were under continuous intravenous sedation-analgesia and muscle paralysis and ventilated with Carestation R860 ventilator (GE Health Care, US) in volume-controlled mode, and PEEP set according to the low PEEP arm of the PEEP/F_I_O_2_ table used in the ARMA trial [2].

### Measurements

Paw, flow and EELV [18] were measured. Pes and gastric pressure (Pga) were recorded with Nutrivent device (Sidam, Italy) after verification of correct placement [19] and non-stress minimal volume implementation [20]. Electrical impedance tomography (EIT) was recorded with the Swisstom BB^2^ monitor (Swisstom AG, Switzerland). Paw, Pes, Pga and flow signals were recorded at 200 Hz with Biopac150 device and Acknowledge software (Biopac inc., US).

### Protocol

Protocol consisted of the following steps (Additional file 1: Figure S2):

1. Supine head-up at 30° at baseline PEEP (PEEP/F_I_O_2_ table).
2. Supine head-up at 30° at Pes-guided PEEP. PEEP was titrated by 1 cmH_2_O-steps to reach P_L_,ee of 3 ± 2 cmH_2_O. This average value was selected because it falls in the middle of the range of PL,ee values used to set PEEP from the Pes-guided strategy in the Epvent 2 trial [9].

After measurements, baseline PEEP was resumed and patient turned to PP with bed inclination between 0° and 15°.

3. PP at baseline PEEP. Same PEEP and ventilator settings as in step 1 were applied.
4. PP at Pes-guided PEEP. PEEP was titrated in prone in same way as in step 2.

For steps 1–4, measurements were done 1 h after change in PEEP.

5. Late Prone. Patients remained in PP for a total of 16 h during which they were randomly allocated into baseline PEEP or Pes-guided strategy. Measurements were done at session end.

### Measurements

At each step, arterial blood gas and EELV were determined. While Paw, Pes, Pga, flow and EIT signals were continuously recorded, a 3-s inspiratory hold followed by a 3-s expiratory hold was performed (Additional file 1: Figure S3).

### Data analysis

P_L_,ee was equal to static end-expiratory pressure of respiratory system (Paw,ee) minus Pes,ee. Static end-inspiratory transpulmonary plateau pressure was computed in two ways [21]: first as P_L_,ei = Paw,ei − Pes,ei (static end-inspiratory plateau pressure of respiratory system minus static end-inspiratory absolute plateau Pes) and second as P_L_,ei_Elastance derived = Paw,ei times lung to respiratory system static elastance ratio (Est,L/Est,rs). Driving pressures of respiratory system (DPrs) and chest wall (DPcw) were equal to Paw,ei-Paw,ee and Pes,ei-Pes,ee, respectively. Transpulmonary driving pressure (DP_L_) was equal to DPrs-DPcw. Est,rs, Est,L and chest wall elastance (Est,cw) were computed as usual.

EIT-derived regional respiratory system compliance was determined [22].

Recruited lung volume elicited by the change in PEEP in the early stage of the study in SP and in PP was computed according to the method described by Dellamonica et al. [23].

### Statistical analysis

Continuous variables were expressed as mean ± standard deviation unless otherwise stated, and categorical variables as count (percentage point). Sample size was computed to 28 based on primary endpoint (Pes,ee) [24]. To account for incomplete data, we planned to include 38 patients. Data were compared by repeated measures 2-factor ANOVA. Categorical variables were compared using chi-squared test. Correlations between continuous variables were performed with Pearson test. Statistical significance level was set to *p* value < 0.05.

## Results

### Patients

From January 1, 2016, to March 31, 2017, 38 patients (27 male) were enrolled (Additional file 1: Table S1 and Figure S1). Each PEEP strategy was applied to 19 patients for the rest of the PP session (Additional file 1: Table S1). Fourteen patients died during the ICU stay (37%). All the data were lacking for Pes-guided PEEP in SP in one patient, who required immediate PP, and in late prone in 2 patients (1 death and 1 patient returned in SP for urgent coronarography). EIT data were not available in 11 patients.

### Effect of Pes-guided PEEP in supine position

**Fig. 1:**
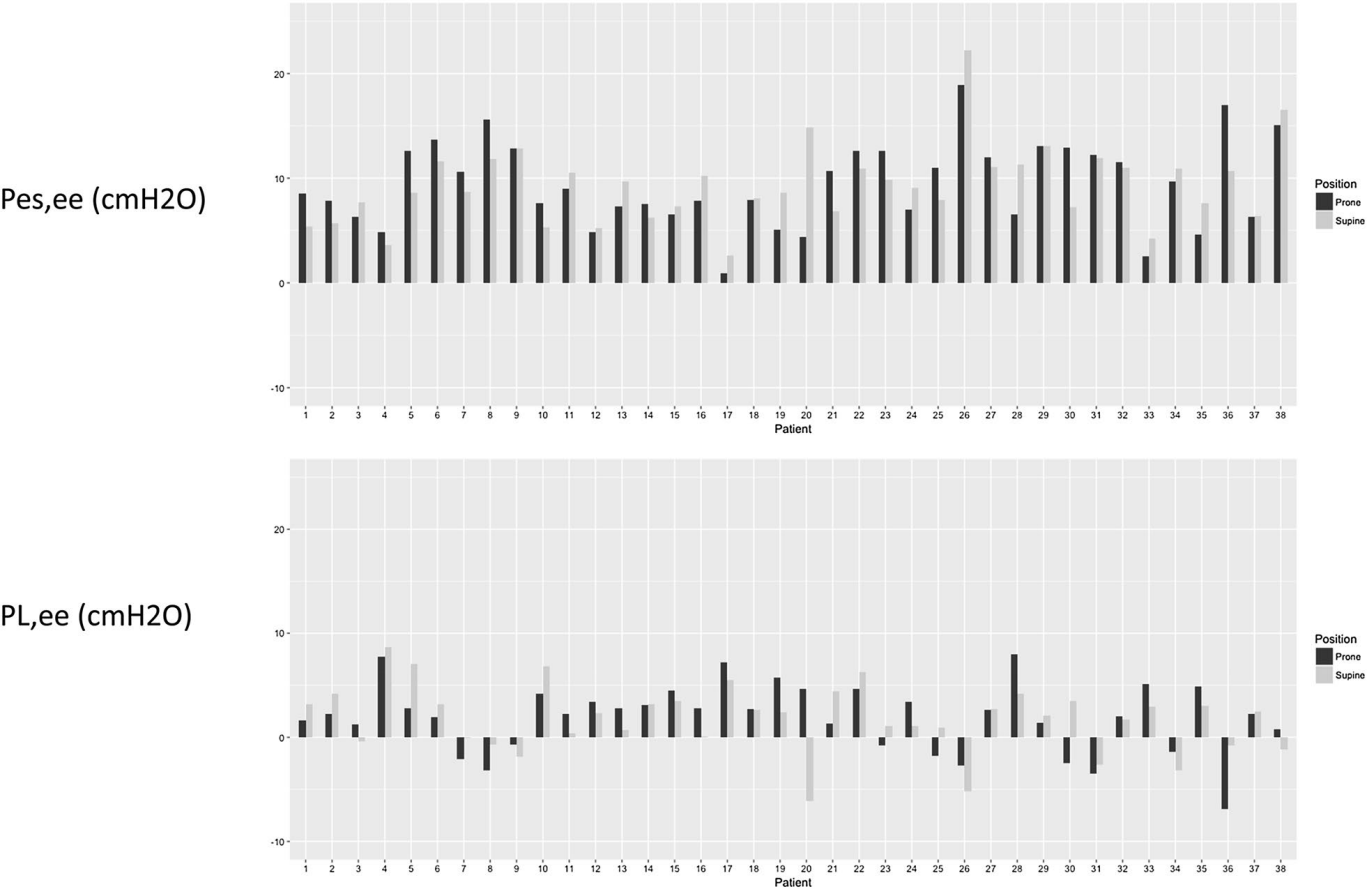
Individual values of static end-expiratory esophageal (Pes,ee) (upper panel) and transpulmonary (PL,ee) (lower panel) pressures in supine and prone positions

**Fig. 2:**
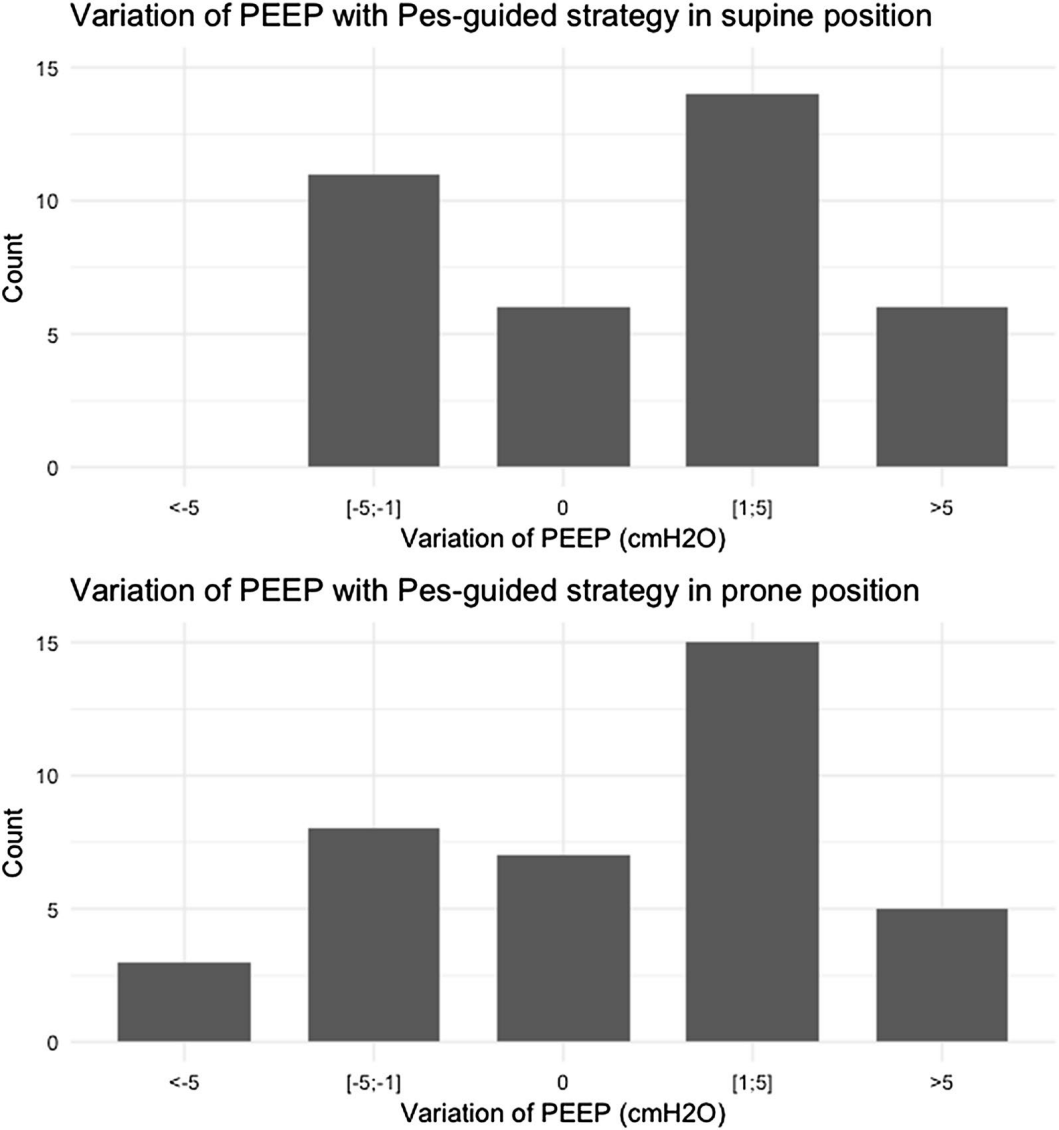
Variation of positive end-expiratory pressure (PEEP) with esophageal pressure (Pes)-guided strategy in supine and prone position

At 10 ± 2 cmH_2_O PEEP set from PEEP/F_I_O_2_ table in SP (step 1), Pes,ee and P_L_,ee averaged 9 ± 4 cmH_2_O and 2 ± 3 cmH_2_O, respectively (Table 1 and Fig. 1). Pes,ee as well as Pga,ee (but not P_L_,ee) correlated to patient’s body mass index: *R* = 0.43 (95% confidence interval [CI] [0.12–0.66]) (*P* = 0.008) and 0.52 [0.25–0.75] (*P* = 0.002), respectively. Ten patients (26%) had negative P_L_,ee. Figure 1 displays the individual values of Pes,ee and P_L_,ee at PEEP set from PEEP/F_I_O_2_ table. PEEP level resulting from the Pes-guided strategy (step 2) averaged 12 ± 4 cmH_2_O (*P* = 0.05 for PEEP strategy). With this strategy, PEEP increased in 20 patients, decreased in 11 patients and did not change in 6 patients (Fig. 2). It was associated with increasing Pes,ei, Pes,ee, and Est,cw (Table 1) and no change in regional lung compliance (Additional file 1: Table S2). Change of PEEP from Pes-guided strategy and body mass index did not correlate.

**Table 1:** Respiratory mechanics in supine and prone position according to PEEP strategy

Variables	Supine position		Prone position		*P* values			N*
	PEEP/F_I_O_2_ table	Pes-guided strategy	PEEP/F_I_O_2_ table	Pes-guided strategy	Position effect	PEEP strategy effect	Position and PEEP interaction	
PEEP set on ventilator (cmH_2_O)	10 ± 2	12 ± 4	10 ± 2	12 ± 5	0.96	0.05	0.96	37
Paw,ee (cmH_2_O)	11 ± 3	13 ± 4	11 ± 3	13 ± 5	0.47	0.05	0.91	37
Paw,ei (cmH_2_O)	23 ± 4	24 ± 6	22 ± 4	24 ± 6	0.11	0.08	0.78	37
Pes,ee (cmH_2_O)	9 ± 4	10 ± 5	9 ± 4	10 ± 5	0.88	0.05	0.83	37
Pes,ei (cmH_2_O)	12 ± 4	14 ± 5	13 ± 4	14 ± 6	0.66	0.01	0.80	37
Pga,ee (cmH_2_O)	12 ± 6	12 ± 5	18 ± 6	18 ± 7	< 0.001	0.19	0.06	31
Pga,ei (cmH_2_O)	14 ± 6	14 ± 6	20 ± 6	21 ± 7	< 0.001	0.11	0.04	31
P_L_,ee (cmH_2_O)	2 ± 3	2 ± 2	2 ± 3	3 ± 2	0.78	0.15	0.96	37
P_L_,ei (cmH_2_O)	10 ± 5	11 ± 5	10 ± 5	10 ± 4	0.03	0.47	0.66	37
P_L_,ei_Elastance (cmH_2_O)	16 ± 5	17 ± 6	15 ± 5	15 ± 6	< 0.01	0.46	0.34	37
DPrs (cmH_2_O)	12 ± 4	12 ± 4	11 ± 3	11 ± 4	< 0.001	0.88	0.65	37
DPcw (cmH_2_O)	3 ± 1	3 ± 2	3 ± 1	4 ± 1	0.33	0.03	0.42	37
DP_L_ (cmH_2_O)	9 ± 4	8 ± 4	8 ± 4	7 ± 4	< 0.001	0.34	0.38	37
Est,rs (cmH_2_O/L)	34 ± 14	35 ± 14	32 ± 14	33 ± 14	0.04	0.46	0.83	37
Est,cw (cmH_2_O/L)	9 ± 4	10 ± 5	10 ± 3	11 ± 4	0.22	0.01	0.30	37
Est,L (cmH_2_O/L)	25 ± 15	25 ± 15	22 ± 15	22 ± 15	0.01	0.71	0.64	37
PaCO_2_ (mmHg)	53 ± 16	55 ± 17	52 ± 14	52 ± 18	0.08	0.12	0.36	36
PaO_2_/F_I_O_2_ ratio (mmHg)	143 ± 28	149 ± 31	172 ± 51	187 ± 53	< 0.001	0.03	0.17	36
EELV (mL)	1359 ± 503	1427 ± 456	1266 ± 391	1328 ± 415	0.01	0.08	0.93	37

Values are mean ± SD

*PEEP* positive end-expiratory pressure, *Paw,ee* static end-expiratory pressure of the respiratory system, *Paw,ei* static end-inspiratory pressure of the respiratory system, *Pes,ee* static end-expiratory esophageal pressure, *Pes,ei* static end-inspiratory esophageal pressure, *Pga,ee* static end-expiratory gastric pressure, *Pga,ei* static end-inspiratory gastric pressure, *P*_*L*_*,ee* static end-expiratory transpulmonary pressure, *P*_*L*_*,ei* static end-inspiratory transpulmonary pressure, *P*_*L*_*,ei _Elastance* Paw,ei × Est,L/Est,rs, *Est,rs*, *Est,cw*, *Est,L* static elastance, of respiratory system, chest wall and lung, respectively, *DPrs, DPcw, DPL* driving pressure of respiratory system, chest wall and lung, respectively, *EELV* end-expiratory lung volume

*Due to incomplete data, some patients were excluded from the analysis

### Effect of prone position and PEEP strategy

By study design, the first PEEP used in PP (step 3) was set from the PEEP/F_I_O_2_ table and, hence was the same as in step 1. At that PEEP applied for 1 h in PP, Pes,ee averaged 9 ± 4 cmH_2_O as in step 1 (*p* = 0.88). There was no effect of position on PEEP level (Table 1), and a trend toward higher PEEP with Pes-guided strategy by 2 cmH_2_O. There was a significant effect of position on lung mechanics at either PEEP (Table 1): P_L_,ei, P_L_,ei_Elastance derived, DP_L_ and Est,L were significantly lower in PP than in SP with no significant interaction with the PEEP strategy (Table 1). On average, P_L_,ee was independent of either PEEP strategy and position. However, for the position, there were individual variations (Fig. 1). In addition, P_L_,ee in step 1 and 3 was correlated: *R* 0.57 [0.31–0.75] (*P* = 0.002). There was a significant effect of PEEP strategy on chest wall mechanics and oxygenation (Table 1). Pga was significantly higher in PP than in SP with no effect of PEEP, but a significant interaction between position and PEEP was observed for Pga,ei (Table 1). Spinal lung compliance, as assessed by EIT, was significantly affected by position at either PEEP with higher values in PP (Additional file 1: Table S2). There was no correlation between variation of Est,cw and variation of PaO_2_/F_I_O_2_ ratio between SP and PP at each PEEP applied.

The recruited lung volume averaged 40 ± 191 and 82 ± 255 ml (*P* = 0.665) in SP and PP, respectively.

### Effect of time in prone position

Over time in PP, EELV and PaO_2_/F_I_O_2_ significantly increased and PaCO_2_ significantly decreased regardless of the PEEP strategy (Table 2). With the Pes-guided PEEP, Est,cw and DPcw were higher than in the PEEP/F_I_O_2_ table group, without effect of time. Over the time spent in PP, we found that sternal lung compliance was higher at the end of the PP session, with no effect of PEEP group (Table 3).

**Table 2:** Respiratory mechanics in early and late prone position according to PEEP strategy

Variables	Early prone position		Late prone position		*P* values		
	PEEP/F_I_O_2_ table group (*n* = 19)	Pes-guided group (*n* = 19)	PEEP/F_I_O_2_ table group (*n* = 19)	Pes-guided group (*n* = 17)	Time effect	PEEP strategy effect	Time and PEEP interaction
PEEP set on ventilator (cmH_2_O)	10 ± 2	12 ± 5	10 ± 2	12 ± 4	0.53	0.23	0.50
Paw,ee (cmH_2_O)	11 ± 3	13 ± 5	11 ± 3	13 ± 4	0.93	0.09	0.26
Paw,ei (cmH_2_O)	22 ± 4	23 ± 5	22 ± 4	24 ± 4	0.79	0.28	0.67
Pes,ee (cmH_2_O)	9 ± 4	10 ± 5	9 ± 4	10 ± 6	0.88	0.47	0.07
Pes,ei (cmH_2_O)	12 ± 5	14 ± 4	12 ± 5	14 ± 5	0.87	0.20	0.07
Pga,ee (cmH_2_O)	18 ± 5	17 ± 6	17 ± 4	17 ± 5	0.48	0.96	0.77
Pga,ei (cmH_2_O)	20 ± 5	20 ± 6	19 ± 4	19 ± 6	0.43	0.96	0.75
P_L_,ee (cmH_2_O)	2 ± 4	3 ± 1	2 ± 4	3 ± 2	0.82	0.22	0.38
P_L_,ei (cmH_2_O)	10 ± 6	9 ± 3	10 ± 5	10 ± 4	0.95	0.69	0.11
P_L_,ei_Elastance (cmH_2_O)	16 ± 5	14 ± 5	15 ± 4	14 ± 5	0.96	0.47	0.16
DPrs (cmH_2_O)	11 ± 4	10 ± 3	11 ± 4	11 ± 4	0.79	0.51	0.17
DPcw (cmH_2_O)	3 ± 1	4 ± 1	3 ± 1	4 ± 1	0.52	0.02	0.42
DP_L_ (cmH_2_O)	8 ± 4	6 ± 3	8 ± 4	7 ± 3	0.82	0.19	0.14
Est,rs (cmH_2_O/L)	34 ± 18	29 ± 11	33 ± 16	30 ± 12	0.76	0.41	0.24
Est,cw (cmH_2_O/L)	9 ± 3	12 ± 4	9 ± 3	11 ± 5	0.59	0.04	0.37
Est,L (cmH_2_O/L)	26 ± 18	18 ± 11	24 ± 16	19 ± 10	0.56	0.18	0.12
PaCO_2_ (mmHg)	50 ± 13	55 ± 13	45 ± 14	48 ± 10	< 0.001	0.42	0.43
PaO_2_/F_I_O_2_ ratio (mmHg)	178 ± 55	193 ± 48	223 ± 85	218 ± 65	< 0.001	0.82	0.27
EELV (mL)	1341 ± 440	1342 ± 445	1504 ± 547	1691 ± 950	0.01	0.63	0.28

Values are mean ± SD

*PEEP* positive end-expiratory pressure, *Paw,ee* static end-expiratory pressure of the respiratory system, *Paw,ei* static end-inspiratory pressure of the respiratory system, *Pes,ee* static end-expiratory esophageal pressure, *Pes,ei* static end-inspiratory esophageal pressure, *Pga,ee* static end-expiratory gastric pressure, *Pga,ei* static end-inspiratory gastric pressure, *P*_*L*_*,ee* static end-expiratory transpulmonary pressure, *P*_*L*_*,ei* static end-inspiratory transpulmonary pressure, *P*_*L*_*,ei _Elastance* Paw,ei x Est,L/Est,rs, *Est,rs, Est,cw, Est,L* static elastance, of respiratory system, chest wall and lung, respectively, *DPrs, DPcw, DPL* driving pressure of respiratory system, chest wall and lung, respectively, *EELV* end-expiratory lung volume

**Table 3:** Regional compliance in early and late prone position according to PEEP strategy

Electrical impedance tomography-derived regional compliance	Early prone position		Late prone position		*P* values		
	PEEP/F_I_O_2_ table (*n* = 13)	Pes-guided strategy (*n* = 10)	PEEP/F_I_O_2_ table (*n* = 13)	Pes-guided strategy (*n* = 10)	Position effect	PEEP strategy effect	Position and PEEP interaction
Sternal lung regions (mL/cmH_2_O)	11 ± 4	13 ± 6	13 ± 6	15 ± 8	0.002	0.42	0.89
Spinal lung regions (mL/cmH_2_O)	22 ± 9	24 ± 9	21 ± 9	24 ± 12	0.99	0.57	0.51

Values are mean ± SD

*PEEP* positive end-expiratory pressure, *Pes* esophageal pressure

## Discussion

The two main findings of the present study that systematically assessed the Pes-guided strategy in PP in ARDS patients were that: (1) PP had no impact on absolute Pes measurements, suggesting the accuracy of esophageal balloon manometry independent of the mass of the mediastinum if esophageal balloon was calibrated properly; (2) PP was effective to improve lung mechanics (immediate effect) and facilitate lung recruitment (slow effect) independent of PEEP levels.

### Impact of PP on Pes measurements

The Pes-guided PEEP concept is primarily driven by Pes,ee. Contrary to our expectations, Pes,ee did not decrease in PP from SP at same PEEP. One explanation may be that in PP pericardial ligaments prevent compression of esophagus by the heart and mediastinum and, hence avoid any real compression onto the esophageal balloon. Another explanation may be that we compared SP30° to PP0°–15°. Pes,ee decreased by 2 cmH_2_O between SP0° and PP0° in ARDS patients [25], as in normal subjects experiencing spine surgery [26].

Since average Pes,ee did not change significantly between SP and PP, the PEEP level resulting from the Pes-guided strategy was the same in both positions and it was systematically 2 cmH_2_O above the amount of PEEP from the PEEP/F_I_O_2_ table. This small change can be due to the fact that P_L_,ee, set from PEEP/F_I_O_2_ table, was near our target.

Talmor et al. [8] reported an average 7 cmH_2_O rise of PEEP with an improvement in oxygenation and Est,rs [8], by using Pes-guided strategy in SP. Potential explanations for the discrepancy between Talmor’s [8] and the present study are: (1) our case mix included mostly primary ARDS; (2) we set a fixed P_L_,ee (3 ± 2 cmH_2_O) goal and did not use a P_L_,ee-F_I_O_2_-table; (3) the correct placement of the esophageal balloon was assessed by the Baydur maneuver, and minimal non-stress esophageal balloon volume was determined; (4) Pes,ee averaged 9 cmH_2_O in our study versus 17 cmH_2_O in Talmor et al’ study [8], for PEEP of 10 and 13 cmH_2_O, respectively.

### Impact of PP on chest wall mechanics

Between SP and PP in our study, Est,cw did not change. This result differs from Mentzelopoulos et al. [24], who found an increase in Est,cw by about 5 cmH_2_O/L between SP60° and PP0°. This discrepancy may be explained by different angulations in SP, and higher *V*_T_ and PEEP in their study, making the volume–pressure curve of the chest wall displaced upward. Indeed, between SP and PP, EELV increased from 1.0 to 1.5 L in Mentzelopoulos et al. study [24] and decreased from 1.4 to 1.1 L in our study. Since the abdominal content has a major influence on the position of the diaphragm, and hence lung volume, SP30° might pull it down while PP might push it upward, which could easily result in the average difference of 0.3 L in lung volume we observed. Pelosi et al. found that chest wall compliance significantly decreased in PP from SP [27]. In two previous studies, we also found an increase in Est,cw in PP [25, 28] at 0° inclination in both positions, as Pelosi et al. [27]. Therefore, the inclination in SP and PP should be taken into account for interpreting the effect of PP on Est,cw.

### Early impact of prone position on lung mechanics

PP significantly improved lung mechanics in the present study independently of PEEP strategy. The decrease in Est,L in PP should indicate lung recruitment or overdistension reduction. Previous CT scan studies found that PP can promote lung recruitment and lessen overdistension [29]. In the present study, EELV did not increase in PP. The effect of PP on EELV did vary across studies from no change [27] to increase [24]. Recently, EELV was found increasing from 1.6 L ± 0.476 to 1.8 ± 0.7 L (*P* = 0.008) after 1 h in PP [18]. In our study, moving the patients from SP30° to SP0° before proning may have significantly decreased EELV so that PP could not improve EELV immediately. Indeed, it took almost 14 h in PP for EELV to surpass its value in SP. The reduction in Est,L in PP could result from an imbalance between recruitment and derecruitment at the regional level with lung recruitment in the spinal parts of the lung being greater than the decrease in aerated lung volume in sternal parts [11]. Our increase in spinal lung compliance in PP favors this hypothesis, even though whole EELV did not change.

DP_L_ [30] should theoretically better reflect lung stress than DPrs [31]. DP_L_ decreased significantly in PP with no effect of PEEP strategy. Therefore, this finding may contribute to the better outcome of patients treated in PP.

P_L_,ei_elastance method may reflect lung stress in the sternal non-dependent parts of the lung in SP [32]. Whether or not PL,ei_Elastance derived in PP still reflects non-dependent parts of the lung or explores the sternal lung region is unknown. We found a significant decrease in P_L_,ei_Elastance derived in PP irrespective of the PEEP strategy. The fact that the compliance of non-dependent lung as assessed with EIT increased in PP suggests that PL,ei_Elastance derived reflects lung stress in that lung region in PP. P_L_,ei was suggested to reflect lung stress in the spinal dependent parts of the lung in SP [32]. Interestingly, P_L_,ei_Elastance derived remained greater than P_L_,ei in both SP and PP.

Taken together, these findings suggest that PP can prevent ventilator-induced lung injury regardless of PEEP strategy. Present results are important because they contribute to explain why survival was significantly improved in the Proseva trial [12] even though low levels of PEEP were used.

### Slow effect of prone position on facilitation of lung recruitment

Over time in PP gas exchange and EELV improved. Increase in EELV may or may not include lung recruitment, defined as a decrease in non-aerated amount of lung tissue, i.e., as lung tissue that regains air. Poorly aerated lung regions that become well aerated can also contribute to higher EELV. The fact that increase in EELV was associated with better gas exchange argues in favor of the recruitment of functional lung tissue over time in PP. The improvement in sternal lung compliance over time in PP suggests a net gain of lung volume in dependent parts of the lung.

### Impact of Pes-guided PEEP strategy on chest wall mechanics

With Pes-guided strategy, lung mechanics did not change but Est,cw increased between PEEP 10 and 12 cmH_2_O, on average, regardless of position or PP duration (Tables 1 and 2). This finding was uncommon in ARDS patients between 10 and 15 cmH_2_O PEEP [27, 33–35] and could be explained by a shift of chest wall volume–pressure curve toward its upper (higher PEEP) or lower (lower PEEP) less compliant parts. Since we did not find a significant increase in EELV with the Pes-guided strategy, we have no clear explanation for this finding. Higher Est,cw makes that Paw dissipates into the chest wall, which could protect the lung from excessive stress and strain.

### Clinical implications

First, in a patient receiving Pes-guided PEEP strategy, it is likely that PEEP in PP will be near that in SP.

Second, as EELV early went down from SP to PP, the PEEP should be increased at this step. On the other hand, if PP promotes lung recruitment over time, higher PEEP should be used after the resumption of proning, i.e., when turning the patient back to SP, to prevent derecruitment [36]. However, whether EELV would decrease after turning patient back to SP at same PEEP was not assessed in the present study.

Third, continuous improvement in oxygenation and EELV over time in PP supports the use of prolonged proning sessions [14].

### Limitations and strengths

Our study is limited by the lack of CT scan or other markers of ventilator-induced lung injury, the lack of EIT data in 11 patients and the not randomized design in the early application of PEEP strategy, which might have resulted in a carry-over effect since each patient was own control. Strengths include proper calibration of esophageal balloon, non-stress balloon volume implementation and detailed description of lung and chest wall mechanics in SP and PP with updated methodology.

## Conclusions

There was no impact of PP on Pes measurements. PP had an immediate improvement effect on lung mechanics and a late lung recruitment effect independent of PEEP strategy.

## Additional file

**Additional file 1: Table S1.** Characteristics, ventilator settings, respiratory mechanics and gas exchange at the time of inclusion of 38 ARDS patients allocated into two PEEP strategies for the rest of the proning session. **Table S2.** Regional compliance in supine and prone position according to PEEP strategy. **Figure S1.** Flow chart of the patients. **Figure S2.** Steps of the protocol. PEEP, positive end-expiratory pressure; Pes, esophageal pressure. **Figure S3.** From top to bottom for each panel, tracings of airway pressure (Paw), esophageal pressure (Pes), gastric pressure (Pga), transpulmonary pressure (PL), and flow over time in patient #13 receiving PEEP/FIO2 table in supine position 30° inclination (A) and in prone position. The first vertical arrow is for end- expiratory occlusion (EEO) and the second for end- inspiratory occlusion (EIO). Same scale for corresponding signals in panels A and B.

## Abbreviations

ARDS: acute respiratory distress syndrome
DPcw: driving pressure of the chest wall
DPL: driving pressure of the lung
DPrs: driving pressure of the respiratory system
EELV: end-expiratory lung volume
EIT: electrical impedance tomography
Est,cw: static elastance of the chest wall
Est,L: static elastance of the lung
Est,rs: static elastance of the respiratory system
FIO_2_: inspired oxygen fraction in air
Paw: airway pressure
Paw,ee: static end-expiratory airway pressure
Paw,ei: static end-inspiratory airway pressure
Pes: esophageal pressure
Pes,ee: static end-expiratory esophageal pressure
Pes,ei: static end-inspiratory esophageal pressure
PEEP: positive end-expiratory pressure
Pga: gastric pressure
Pga,ee: static end-expiratory gastric pressure
Pga,ei: static end-inspiratory airway pressure
PL,ee: static end-expiratory transpulmonary pressure
PL,ei: static end-inspiratory transpulmonary pressure
PP: supine position
SP: prone position
VT: tidal volume
