# Supplementary material for: Effects of positive end-expiratory pressure strategy in supine and prone position on lung and chest wall mechanics in acute respiratory distress syndrome
Source: Ann Intensive Care. 2018 Sep 10;8:86. doi: 10.1186/s13613-018-0434-2 (PMC6134472; doi:10.1186/s13613-018-0434-2)
Supplement: Supplementary file 1 — Additional file 1: Table S1. Characteristics, ventilator settings, respiratory mechanics and gas exchange at the time of inclusion of 38 ARDS patients allocated into two PEEP strategies for the rest of the proning session. Table S2. Regional compliance in supine and prone position according to PEEP strategy. Figure S1. Flow chart of the patients. Figure S2. Steps of the protocol. PEEP, positive end-expiratory pressure; Pes, esophageal pressure. Figure S3. From top to bottom for each panel, tracings of airway pressure (Paw), esophageal pressure (Pes), gastric pressure (Pga), transpulmonary pressure (PL), and flow over time in patient #13 receiving PEEP/FIO2 table in supine position 30° inclination (A) and in prone position. The first vertical arrow is for end- expiratory occlusion (EEO) and the second for end- inspiratory occlusion (EIO). Same scale for corresponding signals in panels A and B. [file 13613_2018_434_MOESM1_ESM.docx]

*Study design and population*

A prospective interventional physiologic study was performed in two university-affiliated hospitals medical intensive care unit (ICU) in Lyon (Hôpital de la Croix Rousse), France and Barcelona (Hospital de Sant Pau), Spain.

Adult patients under invasive mechanical ventilation with a moderate-to-severe ARDS (Berlin definition) [[1](#_ENREF_1)] and a PaO_2_/F_I_O_2_ ratio of 150 mmHg or less, tidal volume (V_T_) equal to 6 mL/kg of predicted body weight, PEEP≥5 cmH_2_O, a prone position (PP) session indicated by the clinician in charge and in whom written informed consent was obtained by the next of kin were included.

Exclusion criteria were: contraindication to PP as previously described [[2](#_ENREF_2)] or to esophageal balloon placement (recent surgery, esophageal varices), patient under ECMO, lung transplant, burns over more than 20% of skin, chronic respiratory failure under home mechanical ventilation or oxygen supplementation, underlying disease with expected fatality within one year, decision made of care withdrawal, language barrier, impossibility to obtain consent for judiciary reasons. The protocol (69HCL14_0333) was approved by the ethical committees (2014-A01714-43) on February 24 2015 and by the national agency on drug regulation (150038B-32) on March 31, 2015, and it was registered onto clinicalTrials.gov (NCT02416037).

The flow chart of the patients is shown in figure S1.

*Study protocol*

*Mechanical ventilation standardization*

Patients were under continuous intravenous sedation-analgesia with midazolam and morphine titrated to reach a Ramsay score of 6 [[3](#_ENREF_3)] and paralysis with Cisatracurium besylate administered with a 15 mg loading dose followed by a continuous infusion of 37.5 mg/h. After inclusion, they were switched to Carestation R860 ventilator (GE Health Care) under volume controlled mode with constant inspiratory flow (close to 1 L/s), PEEP and F_I_O_2_ set according to the PEEP/F_I_O_2_ table of the ARMA trial[[4](#_ENREF_4)] (“low PEEP”), tidal volume 6 mL/kg predicted body weight. Respiratory rate was set to maintain pH in the range 7.2 - 7.4 and PaO_2_ was targeted between 55 and 80 mmHg and plateau pressure below 30 cmH_2_O.

*Measurements*

*Measurement devices*

Between the oral end of the endotracheal and the Y-piece of the ventilator, were attached a port to measure Paw connected to a piezo-resistive sensor (Gabarith TM PMSET, Becton Dickinson, Singapore or Validyne, US), a pneumotachograph (Hans-Rudolph 3700, Hans-Rudolph inc., Shawnee, KS, USA) and the device used to measure end-expiratory lung volume (EELV). The devices dead space amounted to 30 mL.

A probe including esophageal and gastric balloons (Nutrivent, Sidam, Italy) was placed to measure esophageal (Pes) and gastric (Pga) pressures. In order to check for the correct placement the Baydur maneuver[[5](#_ENREF_5)] was performed in both supine and prone positions. Minimal non-stress esophageal balloon volume was determined individually according to the pressure-volume curve of the balloon [[6](#_ENREF_6)].

Electrical impedance tomography (EIT) sensor belt was placed around the chest 5 cm above the xiphoid and connected to the Swisstom BB^2^ monitor (Swisstom AG, Lanquart, Switzerland). The signals of Paw, Pes, Pga and flow were sent to Biopac 150 (Biopac inc., Goletta, CA, USA) and acquired at 200 Hz using Acqknowledge 4.0 software (Biopac inc., Goletta, CA, USA). EIT signals were acquired and analyzed with the Ibex software (Swisstom AG, Lanquart, Switzerland).

*Protocol steps*

The protocol was done along the following steps (figure S2):

1. Supine at baseline PEEP (PEEP/F_I_O_2_ table). The patients were in supine position with a thorax angulation of 30° above the horizontal plane. Measurements were done after 1 hour.
2. Supine at Pes-guided PEEP. After completion of the first step, PEEP was titrated by 1 cmH_2_O-steps to reach a static end-expiratory transpulmonary pressure (PL,ee) of 3±2  cmH_2_O (figure S3). This value was selected because it is the median within the PL,ee range in the Epvent 2 trial [[7](#_ENREF_7)]. The other ventilator settings were kept constant but V_T_ could be lowered if static transpulmonary end-inspiratory plateau pressure (PL,ei) was greater than 25 cmH_2_O. Measurements were done after one hour of application of the resulting PEEP. Then, the baseline PEEP was applied and patient turned to PP with 0°-15° inclination from the horizontal plane. Patients were lying on their own mattress, the abdomen not supported.
3. Prone at baseline PEEP. After one hour in PP at same baseline PEEP and ventilator settings as step 1, measurements were done.
4. Prone at Pes-guided PEEP. PEEP was then titrated in PP in the same way as in step 2. One hour stabilization was applied and measurements repeated.
5. Late Prone. Patients remained in PP for a total of 16 hours during which they were randomly allocated to baseline PEEP or Pes-guided strategy group of 19 patients each. Measurements were done at session end. Then, patient was turned back to supine and the study was adjourned.

*Measurements*

At each step 1 to 5, the measurements were performed in the following order. First, blood was drawn from the arterial line for blood gas measurement in the chemistry lab of the hospital. Then Paw, Pes, Pga, flow and EIT signals were continuously recorded. Then, a 3-second inspiratory hold followed by a 3-second expiratory hold was performed at the ventilator. Then, EELV was measured through the nitrogen wash-in washout method by the ventilator.

Patients’ characteristics, SAPS II on admission and SOFA score at the time of inclusion, ARDS stage and risk factors were recorded.

*Data analysis*

VT was measured by integration of the flow-time curve.

From the 3 s P_L_,ee was obtained by subtracting static end-expiratory pressure of the respiratory system (Paw,ee) from Pes,ee. From the 3-sec end inspiratory occlusion, static end-inspiratory pressures (Paw,ei and Pes,ei for the respiratory system and the chest wall, respectively) were obtained (figure S3). Static transpulmonary plateau pressure (P_L_,ei) was obtained by subtracting Paw,ei from Pes,ei. The driving pressures of the respiratory system (DPrs) and chest wall (DPcw) were computed by substracting Paw,ei from Paw,ee and Pes,ei from Pes,ee, respectively. The transpulmonary driving pressure (DP_L_) was equal to DPrs minus DPcw. The static elastances of the respiratory system (Est,rs) and chest wall (Est,cw) were obtained by diving DPrs and DPcw, respectively, by V_T_. Lung static elastance (Est,L) was equal to Est,rs – Est,cw. P_L_,ei was also measured as Paw,ei times Est,L/Est,rs ratio (P_L_,ei_elastance) [[8](#_ENREF_8)].

The EIT-derived regional compliance was computed over 40 consecutive averaged breaths at each step:

Regional respiratory system compliance was calculated as the compliance of the respiratory system (=1/Est,rs) times the ratio of spinal or sternal variation of impedance (∆Z) to the global ∆Z [[9](#_ENREF_9)].

Recruited lung volume elicited by the change in PEEP in the early stage of the study in SP and in PP was computed according to the method described by Dellamonica et al[[10](#_ENREF_10)]. The recruited volume was computed as the difference between PEEP-induced change in EELV and expected minimal volume increase with PEEP from the lowest PEEP. This latter was obtained by the amount of change in PEEP times the respiratory system compliance at the lowest PEEP. In this analysis the lower and higher PEEP may be that obtained from either PEEP strategy.

*Statistical analysis*

Continuous variables were expressed as mean ± standard deviation (SD), unless otherwise stated, and categorical variables as count (percentage-point).

The primary endpoint was Pes,ee. The secondary endpoints were respiratory mechanics-related variables, EELV, PaO_2_, PaCO_2_ and regional ventilation distribution.

The study was powered on the primary end point according to Mentzelopoulos et al [[11](#_ENREF_11)] where the absolute value of Pes,ee was 12.6 ± 3.1 and 8.6 ± 3.6 cm H_2_O in supine and prone, respectively. For this difference in Pes,ee between supine and PP to be significant at 5% and 90% first and secondary risks, respectively, the sample size was computed to 28. To account for incomplete data and withdrawal of consent and protocol modification, we planned to include 38 patients.

Statistical analysis was performed by two-factor ANOVA for repeated measures.

Categorical variables were compared using chi-squared test.

Correlations between continuous variables were performed with the Pearson test.

Statistical significance level was set to p-value <0.05. Statistical analysis was performed using R Statistical Software, version 3.4.1 (R Foundation for Statistical Computing, Vienna, Austria).

Table S1. Characteristics, ventilator settings, respiratory mechanics and gas exchange at the time of inclusion of 38 ARDS patients allocated into two PEEP strategies for the rest of the proning session

| Variables | Overall  N=38 | PEEP/F_I_O_2_ table (n=19) | Pes-guided PEEP (n=19) | P-value |
| --- | --- | --- | --- | --- |
| Age, years | 63±13 | 65±14 | 61+12 | 0.34 |
| Male | 27 (71) | 13 (68) | 14 (74) | 1.00 |
| Body mass index, kg/m^2^ | 27±5 | 25±4 | 30±5 | 0.01 |
| Height, cm | 171±10 | 170±8 | 172±11 | 0.59 |
| Vasopressors use | 28 (74) | 13 (68) | 15 (79) | 0.71 |
| Charlson score | 4±3 | 3±3 | 4±3 | 0.81 |
| SAPS II | 52±17 | 54±18 | 49±16 | 0.39 |
| Admission SOFA score | 8±4 | 9±4 | 8±5 | 0.91 |
| Inclusion SOFA score | 9±4 | 9±3 | 10±4 | 0.79 |
| Renal replacement therapy | 3 (8) | 0 (0) | 3 (16) | 0.23 |
| Origin of the patient |  |  |  | 0.08 |
| Emergency room | 24 (63) | 11 (58) | 13 (68) |  |
| Short stay ward | 10 (26) | 6 (32) | 4 (21) |  |
| Other ICU | 3 (8) | 1 (5) | 2 (11) |  |
| Long stay ward | 1 (3) | 1 (5) | 0 (0) |  |
| Primary ARDS | 33 (87) | 17 (90) | 16 (84) | 1.00 |
| ARDS risk factors |  |  |  | 1.00 |
| Pneumonia | 28 (74) | 14 (74) | 14 (74) |  |
| Aspiration | 4 (11) | 2 (11) | 2 (11) |  |
| Extra-pulmonary sepsis | 3 (8) | 1 (5) | 2 (11) |  |
| Other | 3 (8) | 2 (11) | 1 (5) |  |
| ARDS stage |  |  |  | 0.73 |
| Moderate | 26 (68) | 12 (63) | 14 (74) |  |
| Severe | 12 (32) | 7 (37) | 5 (26) |  |
| Context |  |  |  | 1.00 |
| Surgical | 3 (8) | 2 (11) | 1 (5) |  |
| Medical | 35 (92) | 17 (90) | 18 (95) |  |
| Ventilator settings |  |  |  |  |
| Flow (L/min) | 61±3 | 61±3 | 61±3 | 1.00 |
| Tidal volume (ml) | 382±64 | 373±64 | 391±65 | 0.39 |
| Tidal volume (ml/kg pbw) | 5.9±0.6 | 5.8±0.7 | 5.9±0.5 | 0.49 |
| PEEP (cmH_2_O) | 10±3 | 10±3 | 10±3 | 0.86 |
| Respiratory rate (breaths/min) | 30±6 | 28±6 | 31±5 | 0.12 |
| F_I_O_2_ (%) | 63±15 | 65±13 | 62±16 | 0.59 |
| Ti/Ttot (%) | 28±6 | 28±6 | 29±5 | 0.48 |
| Gas exchange |  |  |  |  |
| PaO_2_ (mmHg) | 74±16 | 73±14 | 75±18 | 0.70 |
| PaO_2_/F_I_O_2_ | 120±23 | 116±27 | 124±19 | 0.31 |
| PaCO_2_ (mmHg) | 45±11 | 44±11 | 46±11 | 0.66 |
| pH | 7.34±0.09 | 7.35±0.08 | 7.33±0.09 | 0.41 |
| Respiratory system mechanics |  |  |  |  |
| Static end-expiratory pressure (cmH_2_O) | 11±2 | 11±3 | 10±2 | 0.80 |
| Static end-inspiratory pressure (cmH_2_O) | 23±4 | 23±5 | 22±4 | 0.79 |
| Compliance (ml/cmH_2_O) | 36±13 | 34±12 | 37±15 | 0.51 |
| Driving pressure (cmH_2_O) | 12±4 | 12±4 | 12±4 | 0.90 |

Definition of abbreviations: SAPS = simplified acute physiology score, SOFA = sequential organ failure assessment, ICU = intensive care unit, ARDS = acute respiratory distress syndrome, pbw = predicted body weight, Ti=inspiratory time, Ttot=total breath duration, PEEP=positive end expiratory pressure

Values are mean±SD and counts (percent-per group) and counts (percentage per group)

Table S2. Regional compliance in supine and prone position according to PEEP strategy

|  | Supine position | | Prone position | |  |  |  |  |
| --- | --- | --- | --- | --- | --- | --- | --- | --- |
|  | PEEP/F_I_O_2_ Table | Pes-guided strategy | PEEP/F_I_O_2_ Table | Pes-guided strategy | Position p-value | PEEP strategy p-value | Interaction p-value | N* |
| Sternal lung regions (mL/cmH_2_O) | 16 ± 8 | 16 ± 8 | 16 ± 8 | 16 ± 7 | 0.99 | 0.19 | 0.89 | 25 |
| Spinal lung regions (mL/cmH_2_O) | 18 ± 6 | 18 ± 7 | 22 ± 9 | 21 ± 9 | 0.01 | 0.69 | 0.05 | 25 |

PEEP= positive end-expiratory pressure, Pes= esophageal pressure

Values are mean±SD

*In 11 eleven patients EIT was not performed. In additional 2 patients some EIT data was lacking, and these patients were excluded from the analysis.

Figure S1. Flow chart of the patients


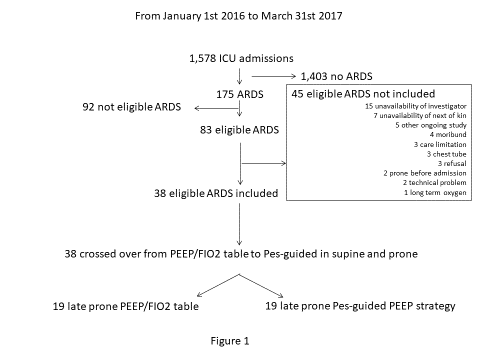


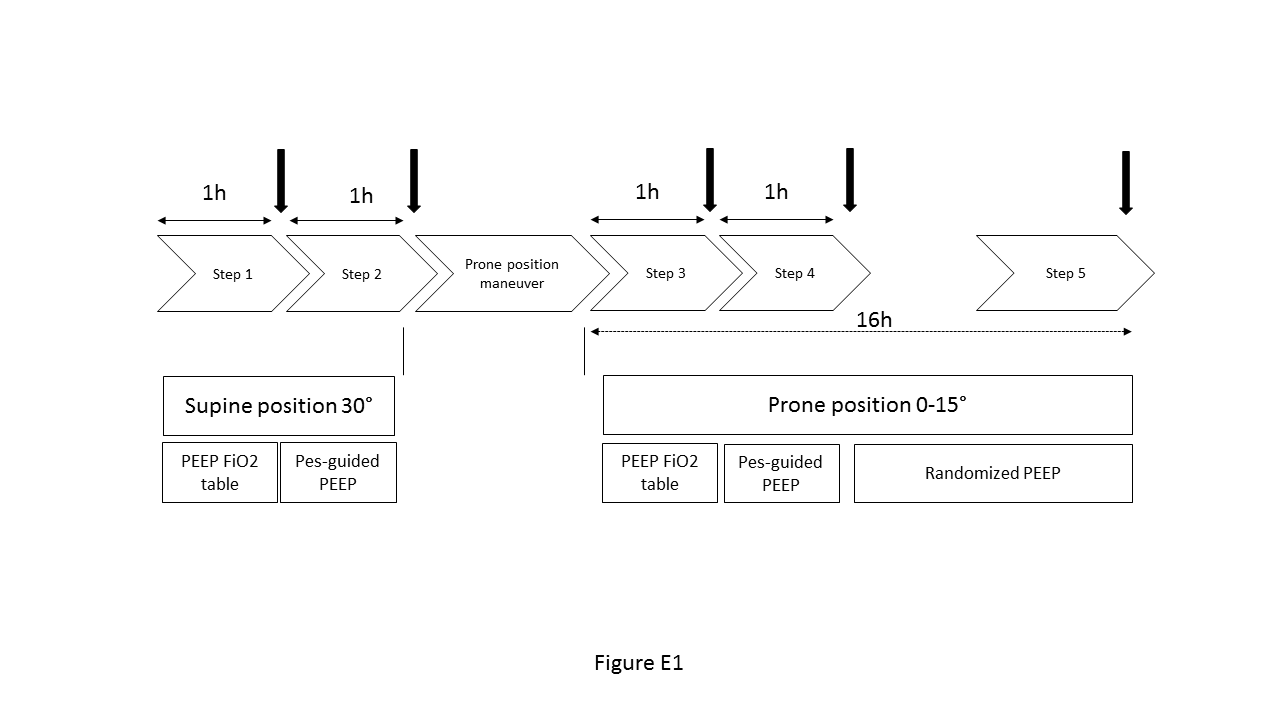


Figure S2. Steps of the protocol. PEEP=positive end-expiratory pressure, Pes=esophageal pressure


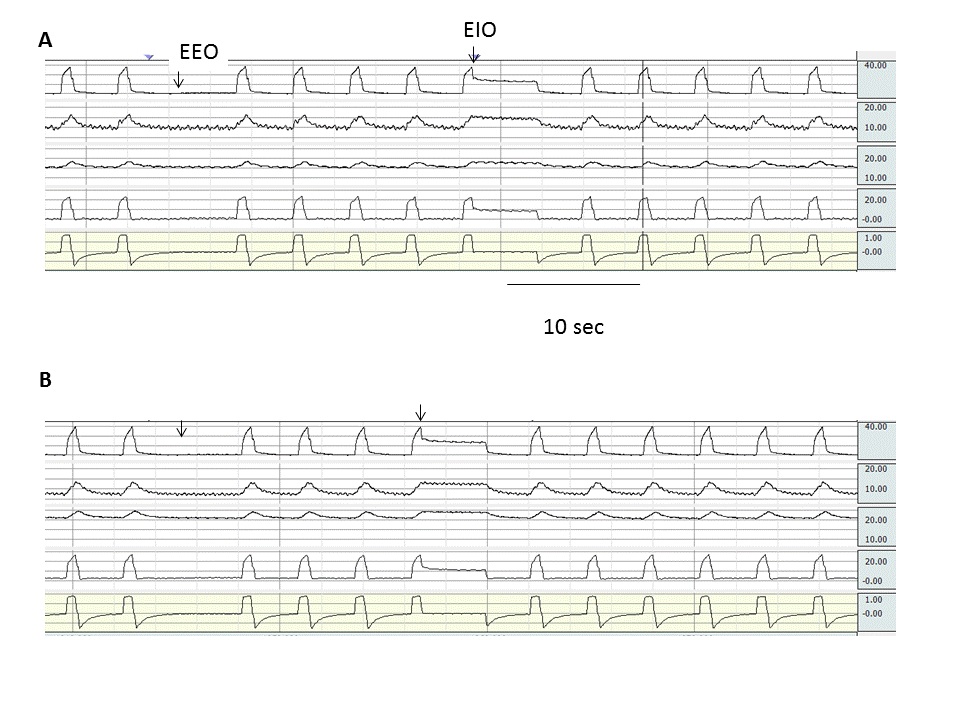


Figure S3. From top to bottom for each panel, tracings of airway pressure (Paw), esophageal pressure (Pes), gastric pressure (Pga), transpulmonary pressure (PL), and flow over time in patient #13 receiving PEEP/FIO2 table in supine position 30° inclination (A) and in prone position. The first vertical arrow is for end- expiratory occlusion (EEO) and the second for end- inspiratory occlusion (EIO). Same scale for corresponding signals in panels A and B.

1. Ranieri VM, Rubenfeld GD, Thompson BT, Fergsuon ND, Cladwell E, Fan E, Camporota L, Slutsky AS, (2012) Acute Respiratory Distress Syndrome. The Berlin Definition. JAMA 307: E1-E8

2. Guerin C, Reignier J, Richard JC, Beuret P, Gacouin A, Boulain T, Mercier E, Badet M, Mercat A, Baudin O, Clavel M, Chatellier D, Jaber S, Rosselli S, Mancebo J, Sirodot M, Hilbert G, Bengler C, Richecoeur J, Gainnier M, Bayle F, Bourdin G, Leray V, Girard R, Baboi L, Ayzac L, Group PS, (2013) Prone positioning in severe acute respiratory distress syndrome. N Engl J Med 368: 2159-2168

3. Ramsay MA, Savege TM, Simpson BR, Goodwin R, (1974) Controlled sedation with alphaxalone-alphadolone. Br Med J 2: 656-659

4. ARDSnet, (2000) Ventilation with lower tidal volumes as compared with traditional tidal volumes for acute lung injury and the acute respiratory distress syndrome. The Acute Respiratory Distress Syndrome Network. N Engl J Med 342: 1301-1308

5. Baydur A, Behrakis PK, Zin WA, Jaeger M, Milic-Emili J, (1982) A simple method for assessing the validity of the esophageal balloon technique. Am Rev Respir Dis 126: 788-791

6. Mojoli F, Iotti GA, Torriglia F, Pozzi M, Volta CA, Bianzina S, Braschi A, Brochard L, (2016) In vivo calibration of esophageal pressure in the mechanically ventilated patient makes measurements reliable. Crit Care 20: 98

7. Fish E, Novack V, Banner-Goodspeed VM, Sarge T, Loring S, Talmor D, (2014) The Esophageal Pressure-Guided Ventilation 2 (EPVent2) trial protocol: a multicentre, randomised clinical trial of mechanical ventilation guided by transpulmonary pressure. BMJ Open 4: e006356

8. Grasso S, Terragni P, Birocco A, Urbino R, Del Sorbo L, Filippini C, Mascia L, Pesenti A, Zangrillo A, Gattinoni L, Ranieri VM, (2012) ECMO criteria for influenza A (H1N1)-associated ARDS: role of transpulmonary pressure. Intensive Care Med 38: 395-403

9. Lowhagen K, Lundin S, Stenqvist O, (2010) Regional intratidal gas distribution in acute lung injury and acute respiratory distress syndrome--assessed by electric impedance tomography. Minerva Anestesiol 76: 1024-1035

10. Dellamonica J, Lerolle N, Sargentini C, Beduneau G, Di Marco F, Mercat A, Richard JC, Diehl JL, Mancebo J, Rouby JJ, Lu Q, Bernardin G, Brochard L, (2011) PEEP-induced changes in lung volume in acute respiratory distress syndrome. Two methods to estimate alveolar recruitment. Intensive Care Med 37: 1595-1604

11. Mentzelopoulos SD, Roussos C, Zakynthinos SG, (2005) Prone position reduces lung stress and strain in severe acute respiratory distress syndrome. Eur Respir J 25: 534-544
